# A pilot randomized clinical trial of gamification to increase medication adherence

**DOI:** 10.1016/j.ahj.2026.107419

**Published:** 2026-03-07

**Authors:** Alexander C Fanaroff, Kayla Clark, Leslie Reid-Bey, Charles Rareshide, Jingsan Zhu, Laurie Norton, Kevin G Volpp

**Affiliations:** a Division of Cardiovascular Medicine, University of Pennsylvania, Philadelphia, PA; b Leonard Davis Institute for Health Economics, University of Pennsylvania, Philadelphia, PA; c Penn Center for Health Incentives and Behavioral Economics, University of Pennsylvania, Philadelphia, PA; d Department of Medical Ethics and Health Policy, University of Pennsylvania, Philadelphia, PA; e Division of General Internal Medicine, University of Pennsylvania, Philadelphia, PA; f The Wharton School, University of Pennsylvania, Philadelphia, PA

## Abstract

**Introduction:**

Medication nonadherence is a key contributor to poor control of cardiovascular risk factors, but most interventions shown to increase adherence are labor-intensive and have not been implemented widely. Gamification interventions informed by behavioral economic theory increase physical activity with minimal cost and personnel requirements. We tested the feasibility of a gamification intervention to increase medication adherence among patients at risk of cardiovascular disease with a history of medication nonadherence.

**Methods:**

Patients seen in a single primary care clinic who were prescribed 1 or 2 antihypertensive medications and a statin and had a recent history of nonadherence were identified and offered enrollment in a pilot randomized controlled trial. Patients were enrolled on the Penn Way to Health platform, provided with a validated home blood pressure cuff, and randomized to attention control or gamification. Attention control patients received daily text messages asking if they took their antihypertensive medication and statin, and biweekly text messages asking them to check and report their blood pressure. Gamification participants received the same text messages, and were also enrolled in a game in which they were provided with 90 points per week and lost 10 points each day they did not report taking their antihypertensive medications or statin and each time they did not report a blood pressure when requested. Each week, participants with 70 points or more moved up a level; those with less than 70 points moved down a level. The intervention continued for 14 weeks, followed by a 4-week post-intervention follow-up period. The trial’s primary outcome was self-reported adherence.

**Results:**

A total of 622 patients were eligible for the study and were contacted by study staff; ultimately 43 (of a planned 84) were enrolled and randomized to gamification (n = 21) or control (n = 22). Mean (SD) age was 65 (7.2), 20 (46.5%) were women, and 25 (58.1%) were Black. Over the 18-week study period, there was no significant difference between arms in adherence to antihypertensive medications (79.6% [gamification] vs. 78.6% [control]; difference between arms, 1.4%, 95% CI −1.2 to 3.9%) or statins (80.4% [gamification] vs. 78.6% [control]; difference between arms, 1.8%, 95% CI −2.2 to 5.9%). There were no differences in self-reported adherence between arms over the post-intervention follow-up period, and similarly no differences between arms in medication adherence by SureScripts data, systolic blood pressure, or low-density lipoprotein cholesterol.

**Conclusions:**

In this pilot randomized controlled trial, we found that a behaviorally-designed gamification intervention did not increase adherence to antihypertensive medications and statins compared with attention control. Challenges with recruiting patients with a history of poor adherence and lack of tools for automated, inexpensive, unobtrusive measurement of daily medication-taking behavior are key limitations to the deployment of gamification to increase medication adherence.

**Clinical trial registration:**

clinicaltrials.gov; unique identifier: NCT05326386

Hypertension and hyperlipidemia have been recognized as risk factors for atherosclerotic cardiovascular disease for more than 60 years,^1^ with dozens of medications approved for treatment, yet control of these risk factors remains poor.^2^ One key contributor to poor risk factor control is medication nonadherence. Among patients with hypertension, > 40% are non-adherent to at least one antihypertensive medication, including > 80% of those with uncontrolled blood pressure.^3^ Similarly, > 40% of patients prescribed statins are non-adherent,^4^ with higher rates of cardiovascular events and death in non-adherent patients.^5^

A recent systematic review found 45 publications of interventions to increase medication adherence among patients with hypertension alone.^6^ In many of these studies, the tested intervention improved medication adherence. However, many of the successful interventions are costly or labor-intensive, and few have been successfully implemented on a population level.^7^ There is therefore a need for scalable approaches to increase medication adherence.

Behavioral economics is a scientific field of inquiry that leverages principles from economics and psychology to both understand and influence how individuals behave.^8–11^ In several recent randomized controlled trials, gamification interventions leveraging key concepts from behavioral economics have improved physical activity in individuals with or at risk for ASCVD.^12–14^ These interventions are low-touch and inexpensive, and successfully adapting them to increase medication adherence would represent a scalable approach to this problem. We therefore conducted a pilot randomized controlled trial of a gamification intervention to increase adherence to antihypertensive medications and statins among patients with a history of nonadherence.

## Methods

### Study design and participants

Gamification and Social Incentives to Augment MEdication Adherence (GAME Adherence) was a randomized clinical trial conducted from September 2022 through September 2023. The trial protocol (Supplemental Methods) was approved by the University of Pennsylvania Institutional Review Board, and all patients provided informed consent for participation and use of their data. Data were not deidentified. The study was conducted using Way to Health,^15^ a research technology platform based at the University of Pennsylvania used to implement and test behavior change interventions. Participants were eligible for the trial if they were ≥ 18 years of age, were prescribed 1 or 2 antihypertensive medications and a statin, had a medication possession ratio (MPR; number of days with a supply of medication divided by total number of days) 40-80% for at least one antihypertensive or statin medication over the past 6 months, and had a primary care provider at a participating Penn Medicine clinic. The upper bound for MPR was selected to ensure that we were enrolling patients who were non-adherent to cardiovascular medications; the lower bound was selected to avoid enrolling patients with more substantial barriers to adherence that may not have been addressed by the gamification intervention. MPR was calculated for each patient using data from Surescripts included in the electronic health record.

### Study procedures

Potentially eligible patients were identified using data from the health system’s clinical data warehouse and were contacted by email, text message, and phone. Each potentially eligible participant received up to 2 emails and up to 3 phone calls from study staff, with each phone call preceded by a text message. Messaging was designed to maximize response to unsolicited outreach based on our team’s experience from previous studies, and noted that the patient’s primary care provider thought they would be a good fit for the study, indicated that the purpose of the study was to identify ways to help patients increase medication adherence, noted that the entire study would be conducted remotely, and included an offer of compensation for participating (up to $100 and a free blood pressure cuff). During the phone calls, participants could agree to participate or decline to participate; if the potentially eligible participant did not answer 3 phone calls, they were assumed to have declined participation. Participants who agreed to participate were provided with a link to the Way to Health platform, where they provided formal consent, enrolled in the study, and completed baseline questionnaires. Study staff then mailed the patient a blood pressure cuff, stickers to place on pill bottles indicating whether the medication was an antihypertensive or statin, and $50. Five days after this shipment, participants received an automated message from the Way to Health platform asking if they had received their blood pressure cuff and had a supply of their cardiovascular medications available. Once participants had a blood pressure cuff and a supply of their medications, the participant was asked by automated text message to check their blood pressure and send it to the study team by return text message. After reporting a blood pressure, the participant was randomized in a 1:1 ratio to control or gamification, using an electronic number generator in the Way to Health platform. Treatment assignment was necessarily unblinded to participants, but participants were not told anything about the other study arm. Investigators, statisticians, and data analysts remained blinded to arm assignments until the analysis was completed.

### Interventions

Participants in both arms received daily automated text messages asking if they took their antihypertensive medications and statins (“Did you take your blood pressure meds today? Text back yes if you did.”) and twice weekly text messages asking them to take their blood pressure and send in the result (“It’s time for your blood pressure check. Please check and send us your blood pressure.”) Participants in the attention control arm received these text messages and no other intervention for 18 weeks.

In the gamification arm, in addition to receiving the same text messages as the control arm, participants were entered into an 14-week game grounded in behavioral economic theory and adapted from gamification interventions that successfully increased physical activity.^12–14^ First, each participant signed a pledge to strive to take their medications each day.^16^ Second, at the start of each week, each participant received 90 points. Each day the participant did not take all of their medications or did not report their home blood pressure as requested (twice weekly), they lost 10 points. When participants took their medications and reported their blood pressure, they retained their points. We chose this “loss-framed” approach because prospect theory shows that highlighting potential losses is more effective at driving behavior change than emphasizing gains.^17^ Third, at the end of each week, participants moved up or down through 5 levels based on their points retained the previous week. Those with 70 points or more advanced one level; those with less than 70 points dropped down a level. Each participant began in the middle level so that they would have immediate motivation to at least maintain this status.^18^ Daily text messages noted the number of points each participant had retained for the week, and weekly text messages informed participants if they moved up or down a level. Fourth, each participant picked a family member or friend to receive an email each week summarizing the participant’s performance, and each participant’s primary care physician received a monthly report about the participant’s blood pressure and medication adherence. Involvement of the patient’s primary care physician and support partners was intended to leverage social accountability, as people typically derive additional motivation from not wanting to disappoint others.

At the end of the study, participants in both arms completed an end-of-study questionnaire, for which they were compensated $25. Participants were also offered an additional $50 compensation to undergo a blood draw for measurement of LDL cholesterol at any approved laboratory.

### Outcome measures

The primary outcome was patient-reported adherence, defined as days taking all medications divided by study days over the entire study duration. This was determined based on patients’ responses to daily text messages asking about adherence; patients who did not respond to the text message were assumed to be nonadherent. Secondary outcomes included change in blood pressure over the study duration, medication possession ratio (MPR) over the study duration and over weeks 14-18, and change in LDL cholesterol from baseline through the end of the study. Blood pressure was measured and reported to study staff by participants twice per week, MPR was captured from the electronic health record using data provided by SureScripts, baseline LDL cholesterol was captured from the electronic health record, and end-of-study LDL was measured by venous blood sample.

### Statistical analysis

*A priori* power calculations were based on data from the WayToText study of bidirectional text messages to improve medication adherence, which closely approximated the design of the control arm of this study.^19^ Consistent with data from this trial, we assumed adherence would be 77% in the control arm with standard deviation of 18%.^19^ The trial was designed to include 84 patients (42 in each arm), providing 80% power to detect a 12 percentage point difference in self-reported adherence between groups, with a two-sided alpha equal to 0.05 and accounting for 15% drop-out or loss to follow-up. The enrollment rate was lower than anticipated, and we ultimately enrolled 43 participants. The study had 57% power to detect a 12 percentage point difference between groups, and 80% power to detect a 16 percentage point difference.

All randomly assigned patients were included in the intention-to-treat analysis.

The primary analysis fit generalized logistic regression models for patient-reported adherence to statins and BP medications on a daily level, adjusting for study arm and with participant random effects. Secondary analyses additionally adjusted for age, sex, race, and adherence during the pre-study period. We repeated these analyses for MPR, modeling this variable by determining whether the participant had or did not have a supply of their BP medication or statin on each day. The output of these models was the odds ratio (OR) for adherence in gamification vs. control; OR > 1 indicates greater adherence in gamification arm, and OR < 1 indicates greater adherence in the control arm. For systolic blood pressure, diastolic blood pressure, and LDL cholesterol, we fit generalized linear regression models, adjusting for study arm, baseline value, and with participant random effects. Secondary analyses additionally adjusted for age, sex, and race (Black vs. not Black). In these models, negative numbers indicate greater blood pressure decrease from baseline in the gamification arm, and positive numbers indicate greater blood pressure decrease in the control arm.

Statistical analyses were performed using SAS version 9.4 (SAS Institute) from October through December 2025.

## Results

**Figure 1.**
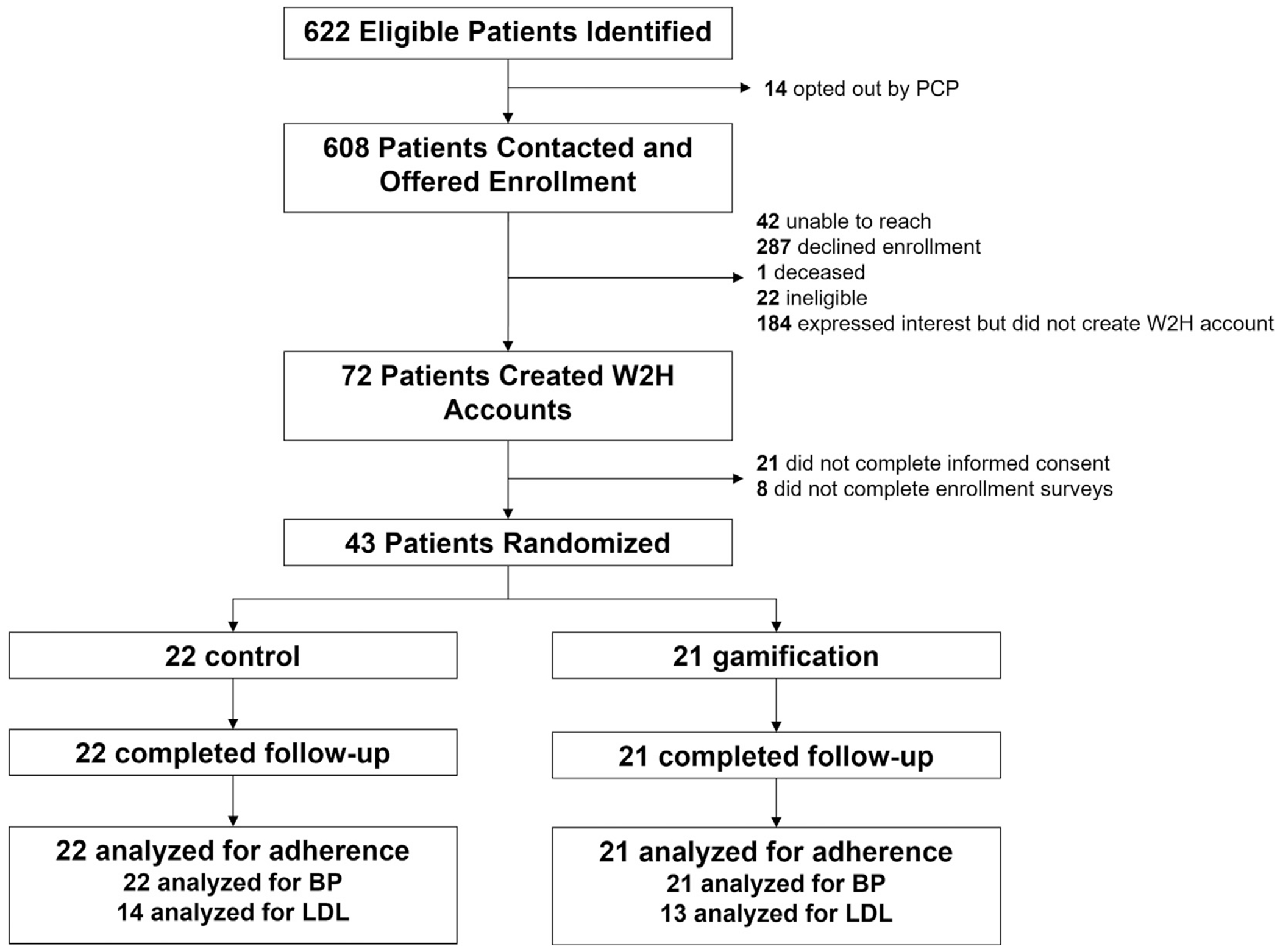
CONSORT diagram. Of 622 eligible participants, 608 were contacted and offered enrollment, 72 began the enrollment process, and 43 were ultimately randomized.

Of 622 eligible participants identified, 608 were contacted and offered enrollment, 72 began the enrollment process, and 43 were ultimately enrolled and randomized to control (n = 22) or gamification (n = 21) (Figure 1). Among 536 patients who did not begin the enrollment process, 287 (53.5%) were able to be contacted but were not interested in participating, 184 (34.3%) expressed some interest but did not proceed to the Way to Health platform to begin enrollment, and 42 (7.8%) could not be reached at all. Demographics and clinical characteristics were similar between the groups (Table 1). Mean (SD) age was 65 (7.2), 20 (46.5%) were women, and 25 (58.1%) were Black. Mean (SD) MPR during the 6 months prior to enrollment was 65.9% (28.1) for statins and 66.4% (25.3) for BP medications. Compared with patients who enrolled in the trial, eligible patients who did not enroll had significantly higher blood pressure (135 vs. 125 mm Hg, p < 0.001) but were otherwise similar (Table S1). In patients who did not enroll in the study, mean (SD) MPR during the 6 months prior to enrollment was 65.1% (28.6) for statins (p = 0.86 compared with enrolled patients) and 62.5% (25.5) for antihypertensive medications (p = 0.34 compared with enrolled patients)

### Medication adherence

**Figure 2.**
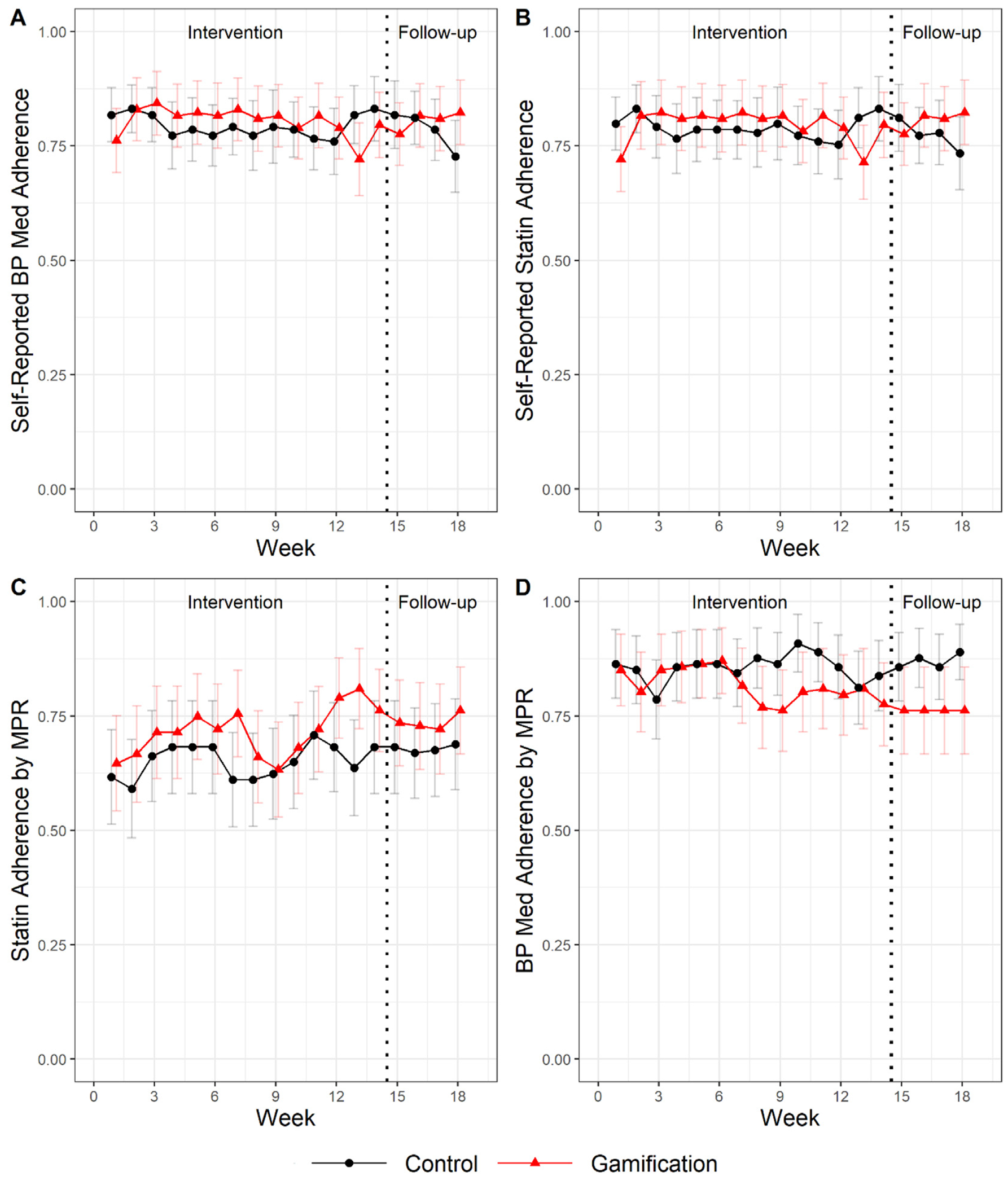
Medication adherence by arm during the intervention and follow-up periods. Panel A shows self-reported adherence to blood pressure medications; Panel B shows self-reported adherence to statins; Panel C shows adherence to blood pressure medications by SureScripts data; Panel D shows adherence to statins by SureScripts data. There were no differences in adherence between arms. Point estimate is mean weekly adherence by arm; error bars represent standard error.

Overall, self-reported medication adherence remained stable throughout the study period (Figure 2a) Throughout the study period, self-reported adherence to antihypertensive medication was 80.4% in gamification and 79.1% in control (difference between arms, 1.4%, 95% CI −1.2 to 3.9%); self-reported adherence to statins was 79.6% in gamification and 78.6% in control (difference between arms, 1.0%, 95% CI −1.6 to 3.5%). During the post-intervention follow-up period, self-reported adherence to antihypertensive medications was 80.4% in gamification and 79.5% in control (difference between arms, 0.9%, 95% CI −3.1 to 5.0%); self-reported adherence to statins was 80.4% in gamification and 78.6% in control (difference between arms, 1.8%, 95% CI −2.2 to 5.9%). Adherence to antihypertensive medications by SureScripts data was 82.0% and 85.7% in gamification and control, respectively, over the study period, and 80.5 and 85.9%, respectively, over the follow-up period (Figure 2b). Adherence to statins by SureScripts data was 71.2% and 64.9% in gamification and control, respectively, over the study period, and 74.2 and 67.9%, respectively, over the follow-up period (Figure 2c and 2d). On the trial’s co-primary outcomes, self-reported adherence to antihypertensive and statin medications during the intervention period, there was no significant differences between arms (OR 1.19, 95% CI 0.22-6.38 for antihypertensive medications; OR 1.04, 95% CI 0.21-5.15 for statin adherence). There were no significant differences in self-reported or SureScripts-defined adherence between the 2 arms over either the intervention or follow-up period (Table 2).

### Blood pressure and LDL-c

**Figure 3.**
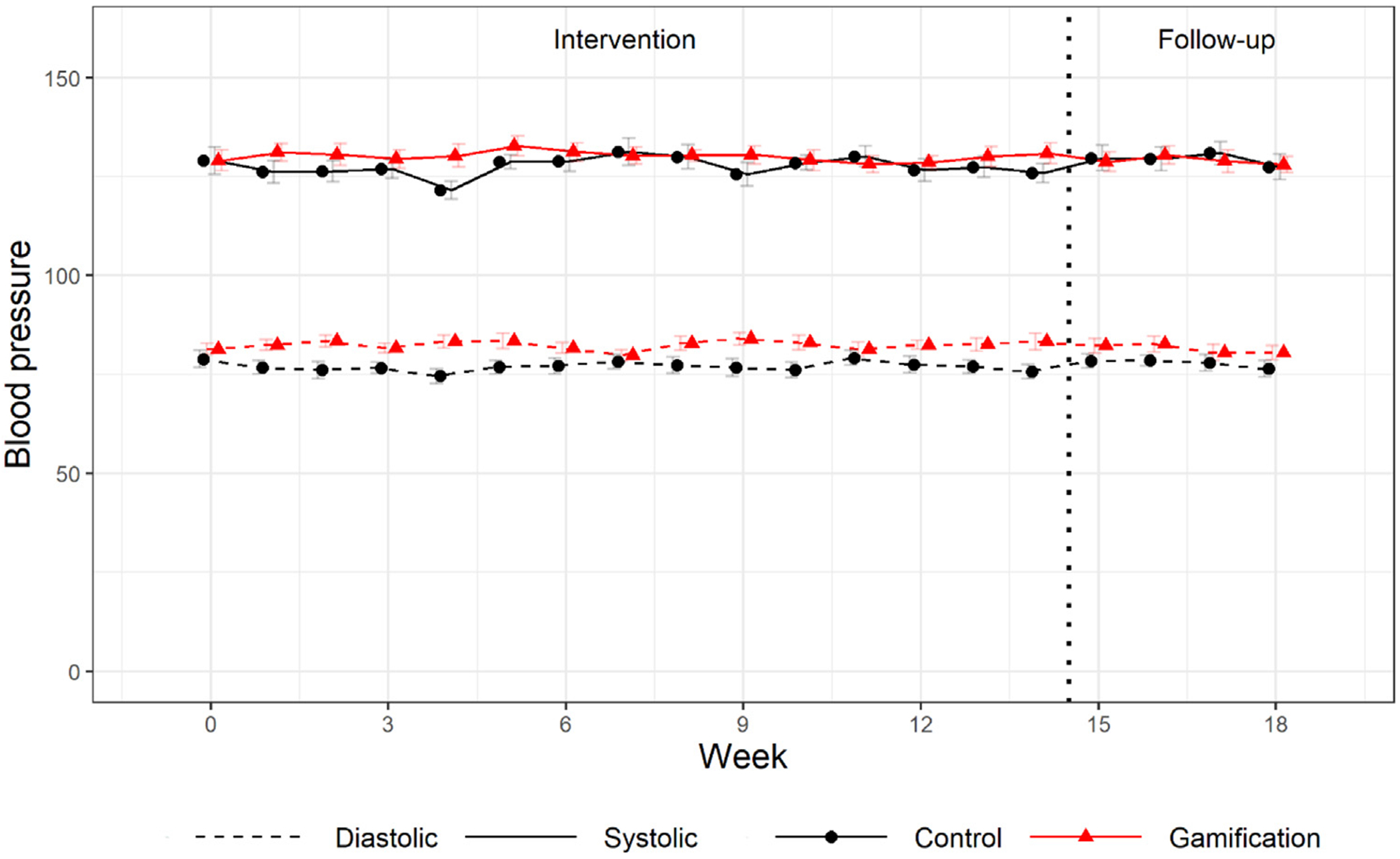
Blood pressure by arm during the intervention and follow-up periods. Systolic and diastolic blood pressure changed minimally in each arm. There was no significant difference between the arms in change from baseline in systolic pressure; the control arm had a greater decrease in diastolic blood pressure over the intervention period. Point estimates are mean weekly systolic and diastolic blood pressure by arm; error bars represent standard error.

Baseline systolic BP, as measured by participants using their home cuff prior to randomization, was 129.9 mm Hg in the gamification arm and 128.4 mm Hg in the control arm. Over the course of the study, at least one BP measurement was submitted on 83.6% of participant weeks in control arm and 83.1% in the intervention arm, and 2 BP measurements were submitted on 68.7% of participant-weeks in the control arm and 72.0% in the intervention arm (Figure S1). In the gamification arm, systolic BP increased by 0.9 mm Hg over the intervention period and 0.7 mm Hg over the follow-up period; in the control arm, systolic BP decreased by 1.6 mm Hg over the intervention period and 1.9 mm Hg over the follow-up period (Figure 3). Diastolic BP was 81.7 mm Hg at baseline in gamification and 78.5 mm Hg in control. It increased by 0.7 mm Hg over the intervention period and decreased by 0.4 mm Hg over the follow-up period in the gamification arm, and decreased by 2.0 mm Hg over the intervention period and 3.1 mm Hg over the follow-up period in the control arm. Over the intervention period, systolic BP decreased by 2.9 mm Hg less in gamification than control (95% CI −1.6 to 7.3 mm Hg), a difference that was not statistically significant, and diastolic BP decreased by 4.7 mm Hg (95% CI 1.9 to 7.5) less in gamification than control. There were no significant differences in change from baseline BP over the follow-up period (Table 3).

Baseline LDL-c was available for 40 patients, and follow-up LDL-c was available for 27. Mean (SD) baseline LDL-c was 74.3 (30.9) mg/dl in gamification and 85.4 (36.4) mg/dl in control; mean end of study LDL was 70 (31.5) mg/dl in gamification and 79.9 (51.3) mg/dl in control. There was no significant difference in change from baseline LDL-c between arms (2.3 mg/dl greater decrease in control vs. gamification, 95% CI −27.9 to 23.4).

## Discussion

In this pilot randomized controlled trial, we found no effect of a gamification intervention, on top of daily text message reminders plus a free home blood pressure cuff, on self-reported or pharmacy fill-based adherence to antihypertensive medications or statins. Similarly, the intervention had no effect on change from baseline in systolic blood pressure or LDL-c. Despite a high-touch outreach strategy in which coordinators were able to make telephone contact with > 90% of eligible participants, just 7% of eligible participants ultimately enrolled, and the study did not meet its enrollment target.

In several randomized controlled trials conducted in different patient populations, gamification enhanced by concepts from behavioral economics meaningfully and durably increased physical activity.^12–14,20,21^ In these studies, the gamification intervention leveraged key concepts from behavioral economics, including immediacy bias (humans overvalue short-term costs relative to long-term benefits), prospect theory (humans are affected by losses more than equivalent gains), and status quo bias (humans’ tendency to overweight the negative aspects to making changes as opposed to sticking with the status quo), to drive behavior change. Unlike other strategies to increase physical activity, including supervised exercise therapy and cardiac rehabilitation, gamification can be implemented at scale without the use of specialized clinical personnel, by automating responses to patient-generated data. We hypothesized that a similarly designed gamification intervention would also be effective for increasing adherence to medications that reduce cardiovascular risk, since taking preventative medications is, like physical activity, a behavior that must be repeated daily primarily for the sake of achieving long-term benefits without any tangible short-term rewards. The trial ultimately did not demonstrate the efficacy of gamification for increasing medication adherence, with several lessons emerging that highlight the challenges of using gamification in this way.

First, the trial did not meet recruitment targets—despite extensive outreach from research coordinators that included email, telephone, and text message outreach to each participant on at least 3 occasions—and was underpowered. Just 12% of eligible patients created an account on the study platform, and only 7% ultimately enrolled. Overall, 46% of eligible patients declined participation and 30% did not proceed to the study platform to create an account. As the intervention focused on increasing medication adherence, we sought to enroll patients with a history of poor adherence. These patients are likely to be less connected to clinical care which may be associated with lower interest in clinical trial participation. The trial’s low enrollment rate despite extensive outreach prompted our decision to halt enrollment prior to reaching enrollment targets, as gamification would not be effective at a population level if patients with poor medication adherence are unlikely to participate. A successful gamification intervention to promote medication adherence must have substantially lower barriers to enrollment to promote broad engagement. This may require allowing research or clinical staff to enroll patients in the program using verbal consent alone or automatically enrolling all eligible participants.

Second, though it is possible that a clinical program, which could automatically enroll eligible participants without a requirement for informed consent, would have had higher uptake, any gamification intervention seeking to leverage immediacy bias to increase medication adherence requires daily reporting of medication-taking behavior. Inexpensive wearable fitness trackers allow for passive measurement of daily step count without burdening patients, but no similar solution exists for daily measurement of medication-taking behavior. Self-report, as used in this trial, requires ongoing effort by patients, and the default condition is non-response. This may be particularly relevant in the context of a clinical program with automatic enrollment, as automatically enrolled patients may be less likely to engage than the participants we enrolled in this trial. Automatic daily reporting of medication-taking could be achieved using electronic pill bottles, but these have a number of limitations, including incompatibility for patients who use pill boxes to manage polypharmacy and high costs that have limited widespread deployment.^22^

A number of studies have combined electronic pill bottles with strategies from behavioral economics to increase medication adherence, but none of these interventions have improved cardiovascular outcomes or risk factors. An intervention combining electronic pill bottles with strategies from behavioral economics increased adherence to antihypertensive medications in a recent randomized controlled trial, though there was no effect on blood pressure.^23^ Similarly, daily lottery-based financial incentives contingent on opening an electronic pill bottle and daily text message reminders each increased statin adherence by 13 to 19 percentage points versus control, with no effect on LDL cholesterol.^24–26^ Small guaranteed daily incentives did not increase adherence to aspirin in patients with recent acute coronary syndrome or warfarin in patients with atrial fibrillation, as measured by electronic pill bottles.^27 , 28^ In a large study enrolling patients with recent myocardial infarction, a comprehensive intervention involving lottery-based financial incentives and social support for medication adherence did not significantly increase adherence to statins, aspirin, *β*-blockers, antiplatelet agents as measured by electronic pill bottles versus control, nor did it reduce recurrent vascular events.^29^ In two other large trials, eliminating copayments for cardiovascular medications improved self-reported medication adherence but did not reduce the incidence of cardiovascular events.^30 , 31^ Since this pilot study was intended to test a lower-touch and scalable approach to increasing medication adherence that could be broadly deployed, we opted not to measure daily adherence with electronic pill bottles due to concerns about the scalability of strategies including these devices.

Our trial does have a number of limitations. First, we did not measure participants’ engagement with the intervention, and it is possible that participants deleted or did not read game-related text messages. Were this the case, using a more engaging and graphical interface, such as a dedicated smartphone or web-based application, to deliver the gamification intervention may have had larger effects.^32^ However, participants responded to approximately 80% of text messages sent during the study and follow-up period, indicating that at least 80% of text messages sent were read. Second, our intervention largely targeted daily medication taking, which is just one part of medication adherence, a complex behavior with multifactorial barriers.^33 , 34^ The intervention did not address financial barriers to adherence or concerns about side effects, for example. Third, we did not reach our recruitment targets and the study was underpowered to detect the 12 percentage point increase in medication adherence that we had initially intended to detect. However, the study was adequately powered to detect a 16 percentage point increase in medication adherence, and confidence intervals for the difference between arms exclude an effect on self-reported medication adherence > 6% between arms. Fourth, our primary outcome was patient-reported medication adherence, which is subject to a number of biases. However, there is no gold standard for medication adherence, and higher patient-reported adherence is associated with better cardiovascular outcomes.^35^ Fifth, patients measured and self-reported their own blood pressure without clinical supervision, and the accuracy of these measurements cannot be substantiated. Lastly, LDL-c at follow-up was only measured in the subgroup of patients who volunteered, limiting the strength of conclusions about the effect of the intervention on this outcome.

Despite the failure of the gamification approach tested in this trial to increase medication adherence, reduce blood pressure, or reduce LDL-c compared with attention control, medication nonadherence remains an important clinical problem and insights from behavioral economics remain a powerful tool for affecting patient behavior. As technology advances and it becomes possible for daily medication-taking behavior to be reported automatically with inexpensive and unobtrusive devices, gamification strategies could be re-tested alongside these devices to determine their effect on medication adherence.

## Conclusions

In this pilot randomized controlled trial, we found that a behaviorally-designed gamification intervention did not increase adherence to antihypertensive medications and statins compared with attention control. Challenges with recruiting patients with a history of poor adherence and lack of tools for automated, inexpensive, unobtrusive measurement of daily medication-taking behavior are key limitations to the deployment of gamification to increase medication adherence.

## Supplementary Material

1

Supplementary material associated with this article can be found, in the online version, at doi:10.1016/j.ahj.2026.107419.

## Figures and Tables

**Table 1.** Baseline characteristics.

	Intervention (N = 21)	Control (N = 22)	Overall (N = 43)
Age (mean, SD)	65 (7.1)	65 (7.5)	65 (7.2)
Female sex (n, %)	8 (38.1%)	12 (54.5%)	20 (46.5%)
Race/ethnicity (n, %)			
Black non-Hispanic	13 (61.9%)	12 (54.5%)	25 (58.1%)
White non-Hispanic	7 (33.3%)	9 (40.9%)	16 (37.2%)
Asian	1 (4.8%)	1 (4.5%)	2 (4.7%)
Education (n, %)			
Some high school or less	2 (9.5%)	3 (13.6%)	5 (11.6%)
High school graduate	6 (28.6%)	5 (22.7%)	11 (25.6%)
Some college or Associate’s degree	5 (23.8%)	6 (27.3%)	11 (25.6%)
College graduate	4 (19%)	4 (18.2%)	8 (18.6%)
Graduate or Professional degree	3 (14.3%)	4 (18.2%)	7 (16.3%)
Missing	1 (4.8%)	0 (0%)	1 (2.3%)
Marital status (n, %)			
Single	2 (9.5%)	5 (22.7%)	7 (16.3%)
Married	13 (61.9%)	13 (59.1%)	26 (60.5%)
Other	6 (28.6%)	4 (18.2%)	10 (23.3%)
Annual household income (n, %)			
< $50,000	5 (23.8%)	5 (22.7%)	10 (23.3%)
$50,000-100,000	9 (42.9%)	3 (13.6%)	12 (27.9%)
> $100,000	6 (28.6%)	8 (36.4%)	14 (32.6%)
Missing	1 (4.8%)	6 (27.3%)	7 (16.3%)
Self-reported health status (n, %)			
Very good	2 (9.5%)	4 (18.2%)	6 (14%)
Good	14 (66.7%)	10 (45.5%)	24 (55.8%)
Fair	2 (9.5%)	8 (36.4%)	10 (23.3%)
Poor	3 (14.3%)	0 (0%)	3 (7%)
BMI (mean, SD)	31.9 (7)	29.5 (4.8)	30.6 (6)
BMI ≥ 30 kg/m^2^ (n, %)	12 (57.1%)	9 (40.9%)	21 (48.8%)
Established ASCVD (n, %)	7 (33.3%)	10 (45.5%)	17 (39.5%)
10-year risk of ASCVD event (mean, SD)*	16.5 (10.9)	16.2 (11.1)	16.3 (10.8)
Baseline LDL (mean, SD)	74.3 (30.9)	85.4 (36.4)	80.1 (33.9)
Baseline LDL levels (n, %)			
>100	5 (23.8%)	7 (31.8%)	12 (27.9%)
70-100	5 (23.8%)	6 (27.3%)	11 (25.6%)
<70	9 (42.9%)	8 (36.4%)	17 (39.5%)
Last Hb A1c (mean, SD)	6.8 (1)	6.6 (1.6)	6.7 (1.3)
Baseline SBP (mean, SD)	129.9 (11.8)	128.4 (16.1)	129.1 (14.0)
Baseline DBP (mean, SD)	81.7 (6.3)	78.5 (9.9)	80.1 (8.4)
Diabetes (n, %)	11 (52.4%)	10 (45.5%)	21 (48.8%)
Current smoking (n, %)	3 (14.3%)	3 (13.6%)	6 (14%)
Heart failure (n, %)	2 (9.5%)	2 (9.1%)	4 (9.3%)
COPD (n, %)	3 (14.3%)	2 (9.1%)	5 (11.6%)
Chronic kidney disease EHR (n, %)	2 (9.5%)	3 (13.6%)	5 (11.6%)
Statin MPR prior to enrollment (mean, SD)^‡^	68.8 (29.1)	63.3 (27.6)	65.9 (28.1)
BP medication MPR prior to enrollment (mean, SD) ^‡^	63.1 (28.7)	69.6 (21.5)	66.4 (25.3)

* calculated using the pooled cohort equations.

‡ calculated for the 6-month period prior to enrollment and used to determine eligibility.

BMI, body mass index; ASCVD, atherosclerotic vascular disease; LDL, low-density lipoprotein; HbA1c, glycated hemoglobin; SBP, systolic blood pressure; DBP, diastolic blood pressure.

**Table 2.** Effect of the intervention on adherence to blood pressure medications and statins.

Outcome	Primary model		Secondary model	
	OR (95% CI)	P value	OR (95% CI)	P value
Self-reported BP medication adherence, study period	1.19 (0.22-6.38)	0.84	1.13 (0.18-6.9)	0.90
Self-reported BP medication adherence, follow-up period	1.10 (0.20-6.03)	0.92	1.12 (0.18-7.09)	0.91
Self-reported statin adherence, study period	1.04 (0.21-5.15)	0.96	1.16 (0.20-6.82)	0.87
Self-reported statin adherence, follow-up period	1.24 (0.23-6.69)	0.80	1.40 (0.21-9.24)	0.72
Surescripts-determined BP medication adherence, study period	0.92 (0.08-10.95)	0.95	1.52 (0.11-21.73)	0.76
Surescripts-determined BP medication adherence, follow-up period	0.49 (0.02-9.59)	0.64	1.05 (0.03-41.81)	0.98
Surescripts-determined statin adherence, study period	1.06 (0.06-17.79)	0.97	1.52 (0.09-26.75)	0.77
Surescripts-determined statin adherence, follow-up period	1.74 (0.07-42.45)	0.73	2.01 (0.07-57.16)	0.68

Primary model adjusted for arm and included participant random effect. Secondary model additionally adjusted for age, sex, race (Black vs. not Black), and Surescripts-determined adherence during the 6-months prior to the study. BP, blood pressure; OR, odds ratio, CI, confidence inter val. Odds ratios compare gamification vs. control: OR > 1 indicates greater adherence in gamification arm; OR < 1 indicates greater adherence in control arm.

**Table 3.** Effect of the intervention on self-reported systolic and diastolic blood pressure.

Outcome	Primary model		Secondary model	
	Difference (Gamification – Control)	P value	Difference (Gamification – Control)	P value
Change in systolic blood pressure, intervention period	2.9 (−1.6 to 7.3)	0.21	2.6 (−1.9 to 7.2)	0.26
Change in systolic blood pressure, follow-up period	1.7 (−4.2 to 7.6)	0.57	1.6 (−4.5 to 7.8)	0.60
Change in diastolic blood pressure, intervention period	4.7 (1.8 to 7.6)	0.002	4.7 (1.9 to 7.5)	0.001
Change in diastolic blood pressure, follow-up period	4.0 (−0.2 to 8.1)	0.06	3.8 (−0.5 to 8.1)	0.09

Primary model adjusted for arm and included participant random effect. Secondary model additionally adjusted for age, sex, and race. When comparing differences, negative numbers indicate greater decrease from baseline blood pressure in the gamification arm (i.e., intervention was effective); positive numbers indicate greater decrease from baseline in the control arm.
