# Supplementary material for: A pilot randomized clinical trial of gamification to increase medication adherence
Source: Am Heart J. Author manuscript; Available in PMC 2026 Jun 11. (PMC13259595; doi:10.1016/j.ahj.2026.107419)
Supplement: 1 [file NIHMS2174571-supplement-1.docx]

**ONLINE ONLY SUPPLEMENTAL MATERIALS**

**Supplemental Methods:** Trial protocol

**Supplemental Table 1:** Baseline characteristics of trial participants and non-participants

**Supplemental Figure 1:** Proportion of participants submitting blood pressure measurements by week

**Supplemental Methods:** Trial protocol

**GAME Adherence**

**Gamification and Social Incentives to Augment MEdication Adherence**

Version 1.1

November 19, 2021

1. **Background**
   1. **Prevalence of Atherosclerotic Cardiovascular Disease, Hypertension, Hyperlipidemia, and Impact**

Atherosclerotic cardiovascular disease (ASCVD) is the leading cause of morbidity and mortality in the United States(1). Hypertension and hyperlipidemia have been recognized as risk factors for ASCVD for more than 60 years (2), with dozens of medications approved for treatment, yet control of these risk factors remains poor (3). Contributors to poor risk factor control include poor uptake of and adherence to medications, persistently unhealthy behaviors, and health system-level incentives favoring treatment of acute events rather than prevention. Poor risk factor control leads to cardiovascular events, including myocardial infarction, stroke, heart failure, and death. Use of low cost cardiovascular drugs could reduce cardiovascular events by 62-88% with perfect adherence (4), highlighting the opportunity to reduce ASCVD risk through translation of efficacious treatments to populations.

Poor medication adherence is an important contributor to poor risk factor control. Following an acute coronary syndrome, up to 50% of patients are non-adherent to cardiovascular medications in some studies, with important implications for cardiovascular events and healthcare costs (5–7). Among patients with hypertension, > 40% are non-adherent to at least one antihypertensive medication, including > 80% of those with uncontrolled blood pressure (8). Similarly, > 40% of patients prescribed statins are non-adherent (9), with higher rates of cardiovascular events and death in non-adherent patients (10).

Black people and groups with low socioeconomic status have a higher prevalence of cardiovascular risk factors, are less likely to be appropriately treated, and are disproportionately likely to suffer a preventable death due to cardiovascular causes (11–15). Some studies have shown that adherence to antihypertensive medications and statins are lower among Black people than white people, and among those with low socioeconomic status (16). Interventions specifically targeting improved medication adherence in Black communities and those with low socioeconomic status may therefore improve cardiovascular health in these vulnerable groups.

- 1. **Previous Interventions to Increase Medication Adherence**

A recent systematic review found 45 publications of interventions to increase medication adherence among patients with hypertension alone (17). Interventions included clinician education, patient education, financial or other incentives, involvement of allied health professionals, simplification of treatment regimens, or enhanced monitoring. In many of these studies, the tested intervention improved medication adherence. However, many of the successful interventions are labor- or personnel-intensive, and few have been shown to reduce cardiovascular events in a larger trial or have been successfully implemented on a population level. There is therefore a need for scalable approaches to increase medication adherence.

**1.3 Behavioral Economic Approaches to Increasing Medication Adherence**

Behavioral economics is a scientific field of inquiry that leverages principles from economics and psychology to understand how individuals behave. Insights from behavioral economics have been shown to both better reflect the ‘predictable irrationality’ of humans, and to be effective in designing interventions that bridge the gap between individuals’ desired behaviors and actual behaviors, achieving sustained increases in physical activity and other healthy behaviors (18–21). In several recent randomized controlled trials, interventions leveraging key concepts from behavioral economics have improved physical activity in individuals with or at risk for ASCVD. These interventions are low-touch and inexpensive, and successfully adapting them to increase medication adherence would represent a scalable approach to this problem.

The key concepts from behavioral economics leveraged by these interventions include gamification and social incentives. Gamification is the application of game design elements such as points and levels in non-game contexts. It is commonly used by workplace wellness programs and digital health applications, however, these interventions are not always optimally designed. In interventions that have led to sustained improvements in physical activity, the gamification component was designed with behavioral insights in mind. First, participants signed a precommittment pledge to strive to achieve their goal (22). Second, the gamification intervention leveraged prospect theory and loss framing, by providing patients with a full set of points every Monday, and taking away points each day if they failed to meet their daily step goal target (23). Third, the gamification intervention leveraged the fresh start effect by giving participants a full set points each week (24). Lastly, the gamification intervention leveraged achievable goal gradients, social status, and progression through the game by having patients move up or down through five levels at the end of each week based on the number of points they have accrued that week.

Social incentives are the influences that motivate individuals to change their behaviors based on social ties or connections. Social networks contain many powerful levers to change human behavior including peer support, accountability, altruism, and collaboration (25–28). Since social networks are ubiquitous, approaches that harness social incentives could be scaled at lower cost than personnel-intensive interventions. Gamification can be enhanced by involving supportive partners in the game, leveraging social connections to increase accountability and add motivation.

**1.5 Rationale for the GAME Adherence Study**

Prior studies have shown the utility of gamification and behavioral economics to increase physical activity in a scalable, inexpensive, and low-touch manner. Like exercise and physical activity, medication adherence is a behavior where individuals’ actions do not meet their intentions, and approaches based on insights from behavioral economics may be useful in changing behavior. This study therefore seeks to test whether a gamification intervention enhanced by two social incentives (support and accountability) improves medication adherence.

1. **Study Objectives**

This pilot randomized controlled trial seeks to test the effectiveness of a behavioral economic-enhanced gamification intervention paired with social incentives (support and accountability) to improve medication adherence in patients with hypertension and hyperlipidemia at a single clinic in West Philadelphia.

1. **Study Design**

**3.1. General Overview**

The GAME Adherence study is a two-arm randomized controlled trial aimed at evaluating whether a gamification intervention plus involvement of a supportive partner (social support) and sending reports to physicians (accountability) increases medication adherence in patients with hypertension and hyperlipidemia. The study will randomize 84 patients with hypertension, hyperlipidemia, and a history of poor medication adherence seen in a single Penn Medicine clinic to an 18-week gamification intervention or to attention control text messages alone (see study protocol figure below).


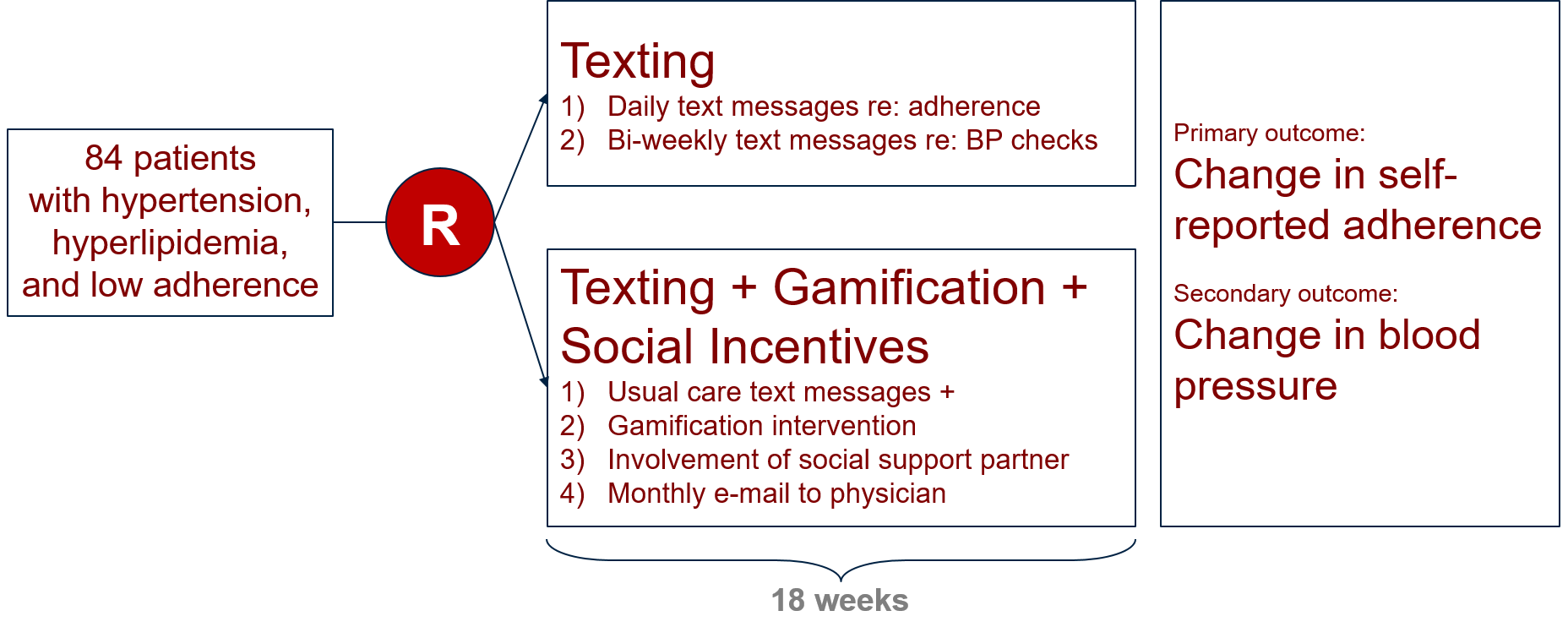


The study will be conducted using Way to Health, a research information technology platform at the University of Pennsylvania used previously for physical activity behavioral interventions. The primary outcome will be self-reported medication adherence. Secondary outcomes will include change in blood pressure over the course of the study and medication possession ratio via pharmacy fill data.

After institutional review board (IRB) approval of the study, patients who meet the eligibility criteria will be contacted by email, phone call or letter to invite them to participate. Those who qualify and express interest will provide online informed consent to the study procedures. All patients will receive blood pressure cuffs and up to $50 to encourage participation and cover costs of the first prescription fill. They will be sent color-coded stickers that they can use to identify their antihypertensive and statin pill bottles. Patients will be texted daily and asked about medication adherence the day before. They will be instructed to measure their blood pressure two times a week and will text these blood pressure results to study staff via the Way to Health platform. All management decisions are completely at the discretion of the care providers. Data capture will include:

- **Baseline case report form** (for each patient) collecting clinical information abstracted from the medical record
- **Daily medication adherence** and **twice weekly blood pressure measurement** captured by bidirectional text messaging
- **Survey and qualitative data** regarding the acceptability of the intervention

**3.2. Site**

GAME Adherence will enroll patients from the Penn Internal Medicine University City practice.

**3.3. Patient Selection Criteria**

**3.3.1. Inclusion Criteria**

Patients are eligible to be included in the study if they are ≥ 18 years of age and meet all of the following criteria:

1. Prescribed 1 or 2 blood pressure medications and a statin medication for > 1 year
2. Has supply of medications at the time of enrollment
3. Medication possession ratio 40-80% for at least one of those medications for the past 6 months
4. Systolic blood pressure > 140 mm Hg on two most recent checks
5. LDL measurement available within the past 12 months
6. Owns a smartphone or tablet operating the iOS or Android operating system

Patients who do not have a supply of their medication will be allowed to complete baseline survey questions but will not be randomized until they certify that they have a supply of all blood pressure medications and statin.

**3.3.2. Exclusion Criteria**

Patients are excluded if they meet any of the following criteria:

1. Unable or unwilling to provide informed consent, including but not limited to cognitive or language barriers (reading or comprehension of English)
2. Systolic blood pressure > 180 mm Hg on either of their last two checks
3. Anticipated life expectancy less than 6 months
4. Any other reason why it is not feasible to complete the entire study

**4. Study Procedures**

**4.1. Screening and Informed Consent**

Patients meeting inclusion criteria will be identified within the electronic health record and Penn Data Store, the health system’s clinical data warehouse. They will be invited to participate by email and will subsequently receive a follow-up text message and phone call. Interested patients will visit the study website on the Way to Health platform to learn more about the study, create an account, provide informed consent, and complete initial baseline eligibility surveys. Study coordinators will be available to assist patients with this process, as necessary.

We will follow an IRB-approved approach taken by many studies using the Way to Health platform to obtaining informed consent. Upon reaching the portal, potential participants will be asked to create an account and will then be informed of the details of the study, including its objectives, duration, requirements, and financial payments. The Way to Health portal will then take interested participants through an online informed consent. The consent document will be divided into sections and potential participants will have to click a button to advance through each section. This is to help ensure that participants read the consent form thoroughly by breaking down the form into manageable blocks of text. Each section will have a button allowing the user to contact a researcher via email or by telephone if they have questions about the consent form. Successive screens will explain the voluntary nature of the study, the risks and benefits of participation, alternatives to participation, and that participants can withdraw from the study at any time. On the final consent screen, potential participants who click a clearly delineated button stating that they agree to participate in the study will be considered to have consented to enroll. Participants will be provided with details regarding how to contact the research team via email or phone at any time if they subsequently wish to withdraw from the study. This contact information will remain easily accessible via the participants’ individual Way to Health web portal dashboards throughout the study.

Support partners will provide verbal informed consent via telephone for their name, email address, and phone number to be stored in the study database.

**4.2. Baseline Questionnaire**

After providing informed consent, participants will complete an online questionnaire to confirm eligibility and complete the study surveys. Patients in both arms will identify a support partner, though only patients ultimately randomized to the gamification and social incentives arm will have their support partners contacted.

The baseline survey will include questions about demographic characteristics and medical history. Patients will also complete several questionnaires to obtain a psychometric profile: the Big Five Inventory survey to evaluate personality characteristics, the PHQ-9 questionnaire to assess mental health state, and the MOS Social Support survey.

After completing the baseline questionnaire, patients will be asked whether they have a supply of their antihypertensive medications and statin. If they have a supply, they will proceed to patient activation. If not, they will be notified that their enrollment has been paused until they obtain a supply of their medications. While enrollment is paused, patients will receive text messages from the study team asking them to certify that they have filled their prescriptions and are ready to begin the study.

**4.3. Patient Activation**

Eligible participants will be mailed a blood pressure cuff and color-coded stickers corresponding to their blood pressure medication(s) and statin. When the stickers arrive, patients will be instructed to apply the stickers to the appropriate pill bottles to help them identify their blood pressure and cholesterol-lowering medications. Five days after their blood pressure cuff and stickers are shipped, they will receive a text message asking if they have received their blood pressure cuffs. Once they have received their blood pressure cuff, they will be activated in the study and will begin receiving daily text messages to track adherence on the following Monday.

**4.4. Randomization**

Once participants are eligible to begin the study, they will be randomly assigned to daily text messages or daily text messages plus gamification and social incentives. Allocation will be 1:1 stratified on number of blood pressure medications with block randomization and block sizes of 2 using an electronic number generator through the Way to Health research technology platform.

**4.5. Study Arms**

**4.5.1 Attention control**

Via the Way to Health platform, all patients will receive daily text messages that ask whether they took their medication the day prior. They will also receive text messages on two days each week asking them to check their blood pressure and report it via return text message.

**4.5.2. Gamification Plus Social Incentives Intervention**

Similar to patients randomized to the attention control arm, patients in the intervention arm will receive daily text messages that ask them whether they did or did not take their medication the day prior, and twice weekly text messages asking them to check their blood pressure and report it via return text message.

They will also be enrolled in a gamification intervention with the following features:

1) Pre-commitment: First, each participant signs a pre-commitment contract agreeing to try their best to take their medications each day. Pre-commitment has been demonstrated to help motivate behavior change.

2) Points: At the start of each week, the participant will receive 90 points (10 for each day of the week plus 10 for each time they are expected to check their blood pressure). Points are endowed rather than given after goal achievement to leverage loss aversion – a concept from prospect theory that reveals that individuals are more motivated by losses than gains. Each day that the patient reports taking his or her medications, and each time that the patient takes his/her blood pressure, they retain their points. However, each time they fail to report taking their medications or checking their blood pressure, they will lose 10 points. Patients will receive daily text messages informing them whether they have lost points for neglecting to take their medication or check their blood pressure the day prior. Points are replenished at the start of the week to leverage the “fresh start effect” – the concept that individuals are more motivated for aspirational behavior around temporal landmarks such as the start of the week.

3) Levels: At the end of the week, if the participant has 70 points or more, they will advance one level. The levels include: blue (lowest), bronze, silver, gold, platinum (highest). If they have less than 70 points, they will drop down one level. This creates a sense of achievable goals (goal gradients) and uses loss aversion to help motivate ongoing efforts to not lose status. Participants will receive weekly text messages informing them of the level (blue, bronze, silver, gold, or platinum) at which they will start the new week, either a promotion or demotion from the week prior. Each participant begins the intervention in the silver level. By starting them in the middle, the higher levels seem within reach and they will feel a sense of loss from dropping down a level in the first week if they don’t achieve 70 points; this may motivate them to initiate greater activity. After 9 weeks, we will reach out to individuals that are stuck in lower levels of blue or bronze and restart them back at silver. This allows for another “fresh start” and creates a new endowment effect as someone who is already at the bottom would otherwise not be able to drop down further and experience loss aversion.

4) Supportive sponsor: Each participant will select a family member or friend of their choice who will serve as a supportive sponsor. This person will receive a weekly email with the participant’s progress including accumulated points, level in the game, and reported medication adherence. This supportive sponsor will help to enhance social incentives to motivate the individual towards his or her goal. We will encourage participants to select someone with whom they come into frequent contact and is close to such as a partner, family member, or friend that they see often. Prior to starting in the study, we will conduct a three-way phone call with the participant and their supportive sponsor. We will provide an overview of study procedures for their arm assignment, and then prompt the participant and supportive sponsor to discuss ways in which they can help the participant adhere to their medication regimen. At least 3 goals will be decided upon during the conversation, and if needed, we will provide suggestions for supporting the participant. The goals that are decided upon will be entered into a survey by study staff, and will be included in weekly emails to the supportive sponsor. After 9 weeks, we will reach out to the participant and supportive sponsor if the participant is stuck in lower levels of blue or bronze to determine if changes should be made to the original support goals. Participants without a supportive sponsor will not be excluded from study participation. Instead, we will identify either a study staff member or peer who has completed the study to serve as their support partner. They will complete a slightly modified version of the baseline and 9-week phone call, if necessary, in two-way conversations with study staff.

5) Accountability: Primary care physicians of patients randomized to the intervention arm will receive a monthly email noting their self-reported adherence and blood pressure. Physicians will not be instructed how to respond to these data, and management will be at their discretion, but participants will know that their physicians are receiving this information.

The intervention will end after 18 weeks.

## **4.6. End-of-Study Questionnaire Completion**

At the end of the 18-week intervention, patients will be alerted by the Way to Health platform to return to the website to complete an end-of-study questionnaire consisting of the questions regarding their experience with the program. The end-of-study questionnaire will also ask whether they are interested in serving as a supportive sponsor for future study participants. Participants who indicate interest will comprise a pool of volunteers from whom peer sponsors will be selected for patients who cannot identify a supportive sponsor. Patients will also receive a text message asking them to go to any approved lab to have their blood drawn to have cholesterol measured. They will be told that having blood drawn is optional, but that if they have it done they will receive $50 compensation.

**4.7. Subject compensation**

Patients will be paid $25 upon completing enrollment to encourage participation and cover costs of first prescription fill, $25 for completing the end-of-study questionnaire, and $50 for having blood drawn at the end of the study.

**4.8 Response to elevated blood pressure readings**

Patients reporting a systolic blood pressure > 180 mm Hg or diastolic blood pressure > 110 mm Hg will be asked if they have symptoms of elevated blood pressure including headache, chest discomfort, shortness of breath, or neurologic changes. If they are asymptomatic, study staff will encourage them to contact their primary care physician to discuss their blood pressure management. If they have symptoms related to elevated blood pressure, study staff will encourage them to visit the emergency room or urgent care, and will simultaneously notify the on-call nurse at the clinic. If the on-call nurse at the clinic cannot be reached, the principal investigator or his designee will be notified.

For other details of safety monitoring, see section 6.5.

# **5. Statistical Methods**

**5.1 Primary and secondary outcome measures**

The study’s primary outcome measure will patient-reported adherence (days taking all medications divided by total days) over the entire study duration.

Secondary outcome measures will include:

1. Change in blood pressure over study duration
2. Patient-reported adherence in weeks 14-18
3. MPR over the entire study period
4. MPR in weeks 14-18
5. Change in LDL from baseline (pre-study) to week 18

**5.2. Sample Size**

Assuming 77% adherence in control arm (WayToText bidirectional testing arm adherence), and SD 18% (Diabetes Care 2013;36:831), we would need to enroll 72 patients to have 80% power to detect a 12% difference between groups with alpha = 0.05. Accounting for 15% drop-out/loss to follow-up, we will enroll 84 patients.

**5.3. Data Analyses**

Data for all consented patients, whether or not they completed all protocol requirements, will be included for analysis. Patient demographics, risk profiles, clinical characteristics, and baseline questionnaire results will be reported. The primary analysis for all outcomes will be a z-test comparing patients randomized to the intervention and control arms. We will use two-tailed p values with p = 0.05 set as the threshold for statistical significance. In exploratory analyses, we will repeat these comparisons using a beta regression model and adjusting for baseline characteristics.

All analyses will be performed using intention-to-treat. By definition, data cannot be missing, as patients will be assumed to be non-adherent on days that they do not report whether they took their medications or do not report a blood pressure. If patients drop out of the study or die during the study, adherence will be calculated only for the time that they participated in the study. LDL will be analyzed only in those patients completing the end-of-study blood draw.

# **6. Human Research Protection**

**6.1 Data confidentiality**

Paper-based records will be kept in a secure location and only be accessible to personnel involved in the study. Computer-based files will only be made available to personnel involved in the study through the use of access privileges and passwords. Wherever feasible, identifiers will be removed from study-related information. Precautions are in place to ensure the data are secure by using passwords and encryption, because the research involves web-based surveys.

**6.2 Subject confidentiality**

Research material will be obtained from participant surveys. All participants will provide informed consent for access to these materials. The data to be collected include data on participant characteristics and behaviors. Research material that is obtained will be used for research purposes only. The same procedure used for the analysis of automated data sources to ensure protection of patient information will be used for the survey data, in that patient identifiers will be used only for linkage purposes or to contact patients. The study identification number, and not other identifying information, will be used on all data collection instruments. All study staff will be reminded to appreciate the confidential nature of the data collected and contained in these databases. The Penn Medicine Academic Computing Services (PMACS) will be the hub for the hardware and database infrastructure that will support the project and is where the Way to Health web portal is based. The PMACS is a joint effort of the University of Pennsylvania's Abramson Cancer Center, the Cardiovascular Institute, the Department of Pathology, and the Leonard Davis Institute. The PMACS provides a secure computing environment for a large volume of highly sensitive data, including clinical, genetic, socioeconomic, and financial information. Among the IT projects currently managed by PMACS are: (1) the capture and organization of complex, longitudinal clinical data via web and clinical applications portals from cancer patients enrolled in clinical trials; (2) the integration of genetic array databases and clinical data obtained from patients with cardiovascular disease; (3) computational biology and cytometry database management and analyses; (4) economic and health policy research using Medicare claims from over 40 million Medicare beneficiaries. PMACS requires all users of data or applications on PMACS servers to complete a PMACS-hosted cybersecurity awareness course annually, which stresses federal data security policies under data use agreements with the university. The curriculum includes Health Insurance Portability and Accountability Act (HIPAA) training and covers secure data transfer, passwords, computer security habits and knowledge of what constitutes misuse or inappropriate use of the server. We will implement multiple, redundant protective measures to guarantee the privacy and security of the participant data. All investigators and research staff with direct access to the identifiable data will be required to undergo annual responsible conduct of research, cybersecurity, and HIPAA certification in accordance with University of Pennsylvania 334 regulations. Data will be stored, managed, and analyzed on a secure, encrypted server behind the University of Pennsylvania Health System (UPHS) firewall. All study personnel that will use this data are listed on the IRB application and have completed training in HIPAA standards and the CITI human subjects research. Data access will be password protected. Whenever possible, data will be deidentified for analysis.

**6.3 Subject privacy**

Interested participants will be directed to the Way to Health portal where they will be asked to enter data related to eligibility and their demographic characteristics. Enrollment will include a description of the voluntary nature of participation, the study procedures, risks and potential benefits in detail. The enrollment procedure will provide the opportunity for potential participants to ask questions and review the consent form information prior to making a decision to participate. Participants will be told that they do not have to answer any questions if they do not wish and can drop out of the study at any time, without affecting their medical care or the cost of their care. They will be told that they may or may not benefit directly from the study and that all information will be kept strictly confidential, except as required by law. Subjects will have access to a copy of the consent document. All efforts will be made by study staff to ensure subject privacy.

**6.4 Data disclosure**

The following entities, besides the members of the research team, may receive protected health information (PHI) for this research study: Greenphire ClinCard, the company which processes study related payments. Patient addresses and account balances will be stored on their secure computers. Twilio, Inc., the company which processes some study-related messages. Twilio will store patients' phone numbers on their secure computers. Qualtrics, Inc., the company which processes most study-related surveys. Qualtrics will house de-identified answers to these surveys on their secure servers. The Office of Human Research Protections at the University of Pennsylvania -Federal and state agencies (for example, the Department of Health and Human Services, the National Institutes of Health, and/or the Office for Human Research Protections), or other domestic or foreign government bodies if required by law and/or necessary for oversight purposes.

**6.5 Data safety and monitoring**

The Principal Investigator will be responsible for monitoring the study. All participants will be given anticipatory guidance on when to seek medical attention. In addition, participants will be asked to report to the study team any injuries or medical care that they feel resulted from participation in the study. They can either call the study team or send an email. The research coordinator will call the participant to collect information regarding the issue and then the PI will review and determine whether it is ok to proceed, further investigation is needed, or the participant should stop the study. For this study there will be no stopping rules or endpoints and thus no planned interim analyses.

**6.6 Risk/benefit**

**6.6.1 Potential study risks**

The major potential risk of this study is a breach of participant confidentiality. We will minimize this risk by using secure data methods as described previously. Due to the financial incentives in this study, we will be collecting social security numbers so that we can complete W-9 forms for participants. Social security numbers only will be used to generate W-9 forms and will be deleted once they are no longer needed. We will also collect home addresses to mail incentive payments. This will be done through a University of Pennsylvania approved partnership with Wells Fargo. Accidental disclosure of social security numbers could lead to identity theft. We will use commercial-grade encryption to protect social security information in transit. Names and addresses will be stored in encrypted databases. These data will be viewable only by the respective participants, the study coordinator(s) and the project manager(s). All other members of the research team will be able to view only participant ID numbers. Even the study arms will be identified by code letters until both the statistician and PI agree that analysis is complete.

There is also a limited potential for risks associated with phlebotomy, a commonly performed procedure that will be performed at certified laboratories, exclusively by individuals specially trained in this procedure. Standard phlebotomy techniques to minimize venipuncture side effects (hand hygiene, glove use, skin antisepsis, and the use of sterile, single-use needles will minimize the risk of infection; appropriate angle of needle entry to avoid hematoma, application of pressure for 3-5 minutes after drawing blood). There is a risk of discomfort, bleeding which is generally mild, bruising, and a remote risk of infection that will be minimized by using these techniques.

**6.6.2 Potential study benefits**

Through participation in this study, each participant will have the potential to increase medication adherence which could improve their health and reduce their risk for future disease. Participants will also receive a free blood pressure cuff and up to $100 for participating. If this approach is effective, it could have tremendous benefits for society if adopted on a wide scale to help individuals. It is expected that other people will gain knowledge from this study and that participation could help understand how to effectively motivate individuals to change behavior. Participants may also receive no benefit from their participation in the study.

**6.6.3 Risk/benefit assessment**

Anticipated risks of this study should be minimal and the risk/benefit ratio is very favorable. To minimize the chance for serious and unexpected adverse events, study participants will be screened through exclusion criteria for any health conditions that may be exacerbated by participating in this study. We have previously outlined the procedures that will be used to prevent a breach of participant data.

# **7.** **References**

1. Cardiovascular diseases (CVDs) [Internet]. [cited 2021 Nov 19]. Available from: https://www.who.int/news-room/fact-sheets/detail/cardiovascular-diseases-(cvds)

2. Kannel WB. Factors of Risk in the Development of Coronary Heart Disease—Six-Year Follow-up Experience: The Framingham Study. Ann Intern Med. 1961 Jul 1;55(1):33.

3. Virani SS, Alonso A, Aparicio HJ, Benjamin EJ, Bittencourt MS, Callaway CW, et al. Heart Disease and Stroke Statistics—2021 Update: A Report From the American Heart Association. Circulation [Internet]. 2021 Feb 23 [cited 2021 Nov 19];143(8). Available from: https://www.ahajournals.org/doi/10.1161/CIR.0000000000000950

4. Lonn E, Bosch J, Teo KK, Pais P, Xavier D, Yusuf S. The Polypill in the Prevention of Cardiovascular Diseases: Key Concepts, Current Status, Challenges, and Future Directions. Circulation. 2010 Nov 16;122(20):2078–88.

5. Chen H-Y, Saczynski JS, Lapane KL, Kiefe CI, Goldberg RJ. Adherence to evidence-based secondary prevention pharmacotherapy in patients after an acute coronary syndrome: A systematic review. Heart Lung. 2015 Jul;44(4):299–308.

6. Rasmussen JN, Chong A, Alter DA. Relationship Between Adherence to Evidence-Based Pharmacotherapy and Long-term Mortality After Acute Myocardial Infarction. JAMA. 2007 Jan 10;297(2):177.

7. Sokol MC, McGuigan KA, Verbrugge RR, Epstein RS. Impact of Medication Adherence on Hospitalization Risk and Healthcare Cost. Med Care. 2005 Jun;43(6):521–30.

8. Abegaz TM, Shehab A, Gebreyohannes EA, Bhagavathula AS, Elnour AA. Nonadherence to antihypertensive drugs: A systematic review and meta-analysis. Medicine (Baltimore). 2017 Jan;96(4):e5641.

9. Ofori-Asenso R, Jakhu A, Zomer E, Curtis AJ, Korhonen MJ, Nelson M, et al. Adherence and Persistence Among Statin Users Aged 65 Years and Over: A Systematic Review and Meta-analysis. J Gerontol Ser A. 2018 May 9;73(6):813–9.

10. De Vera MA, Bhole V, Burns LC, Lacaille D. Impact of statin adherence on cardiovascular disease and mortality outcomes: a systematic review: Impact of statin adherence. Br J Clin Pharmacol. 2014 Oct;78(4):684–98.

11. Van Dyke M, Greer S, Odom E, Schieb L, Vaughan A, Kramer M, et al. Heart Disease Death Rates Among Blacks and Whites Aged ≥35 Years — United States, 1968–2015. MMWR Surveill Summ. 2018 Mar 30;67(5):1–11.

12. Khan MS, Kumar P, Sreenivasan J, Khan SU, Michos ED, Yancy CW, et al. Preventable Deaths From Heart Disease and Stroke Among Racial and Ethnic Minorities in the United States. Circ Cardiovasc Qual Outcomes [Internet]. 2021 Jul [cited 2021 Nov 19];14(7). Available from: https://www.ahajournals.org/doi/10.1161/CIRCOUTCOMES.121.007835

13. Ahluwalia JS, McNagny SE, Rask KJ. Correlates of Controlled Hypertension in Indigent, Inner-City Hypertensive Patients. J Gen Intern Med. 1997 Jan;12(1):7–14.

14. Kotchen JM, Shakoor-Abdullah B, Walker WE, Chelius TH, Hoffmann RG, Kotchen TA. Hypertension control and access to medical care in the inner city. Am J Public Health. 1998 Nov;88(11):1696–9.

15. Shea S, Misra D, Ehrlich MH, Field L, Francis CK. Predisposing Factors for Severe, Uncontrolled Hypertension in an Inner-City Minority Population. N Engl J Med. 1992 Sep 10;327(11):776–81.

16. Lewey J, Shrank WH, Bowry ADK, Kilabuk E, Brennan TA, Choudhry NK. Gender and racial disparities in adherence to statin therapy: A meta-analysis. Am Heart J. 2013 May;165(5):665-678.e1.

17. Pinho S, Cruz M, Ferreira F, Ramalho A, Sampaio R. Improving medication adherence in hypertensive patients: A scoping review. Prev Med. 2021 May;146:106467.

18. Loewenstein G, Brennan T, Volpp KG. Asymmetric Paternalism to Improve Health Behaviors. JAMA. 2007 Nov 28;298(20):2415.

19. Loewenstein G, Asch DA, Volpp KG. Behavioral Economics Holds Potential To Deliver Better Results For Patients, Insurers, And Employers. Health Aff (Millwood). 2013 Jul;32(7):1244–50.

20. Patel MS, Small DS, Harrison JD, Fortunato MP, Oon AL, Rareshide CAL, et al. Effectiveness of Behaviorally Designed Gamification Interventions With Social Incentives for Increasing Physical Activity Among Overweight and Obese Adults Across the United States: The STEP UP Randomized Clinical Trial. JAMA Intern Med. 2019 Dec 1;179(12):1624–32.

21. Patel MS, Benjamin EJ, Volpp KG, Fox CS, Small DS, Massaro JM, et al. Effect of a Game-Based Intervention Designed to Enhance Social Incentives to Increase Physical Activity Among Families: The BE FIT Randomized Clinical Trial. JAMA Intern Med. 2017 Nov 1;177(11):1586–93.

22. Rogers T, Milkman KL, Volpp KG. Commitment devices: using initiatives to change behavior. JAMA. 2014 May;311(20):2065–6.

23. Kahneman D, Tversky A. Prospect Theory: An Analysis of Decision under Risk. Econometrica. 1979;47(2):263–91.

24. Dai H, Milkman KL, Riis J. Put Your Imperfections Behind You: Temporal Landmarks Spur Goal Initiation When They Signal New Beginnings. Psychol Sci. 2015 Dec;26(12):1927–36.

25. Asch DA, Rosin R. Engineering Social Incentives for Health. N Engl J Med. 2016 Dec 29;375(26):2511–3.

26. Christakis NA, Fowler JH. The collective dynamics of smoking in a large social network. N Engl J Med. 2008 May 22;358(21):2249–58.

27. Christakis NA, Fowler JH. The Spread of Obesity in a Large Social Network over 32 Years. N Engl J Med. 2007 Jul 26;357(4):370–9.

28. Jackson SE, Steptoe A, Wardle J. The influence of partner’s behavior on health behavior change: the English Longitudinal Study of Ageing. JAMA Intern Med. 2015 Mar;175(3):385–92.

**Supplemental Table 1:** Baseline characteristics of trial participants and non-participants

|  | **Randomized (n = 43)** | **Not Randomized (n = 579)** | **P value^*^** |
| --- | --- | --- | --- |
| Age | 64 (7.2) | 64 (10.0) | 0.70 |
| Female sex | 20 (46.5) | 312 (53.9) | 0.43 |
| Race |  |  | 0.84 |
| Black | 25 (58.1) | 365 (63.0) |  |
| White | 14 (32.6) | 168 (29.0) |  |
| Asian | 2 (4.7) | 30 (5.2) |  |
| Other | 2 (4.7) | 16 (2.8) |  |
| Hispanic Ethnicity | 0 (0) | 10 (1.7) | 0.43 |
| 10-year ASCVD risk | 16.3 (10.8) | 19.7 (11.9) | 0.15 |
| Smoking status |  |  | 0.66 |
| Current | 5 (11.6) | 81 (14.0) |  |
| Former | 19 (44.2) | 216 (37.3) |  |
| Never | 19 (44.2) | 282 (48.7) |  |
| Established ASCVD | 17 (39.5) | 232 (40.1) | 1.00 |
| Diabetes | 21 (48.8) | 286 (49.4) | 1.00 |
| Heart failure | 4 (9.3) | 96 (16.6) | 0.30 |
| Chronic kidney disease | 5 (11.6) | 123 (21.2) | 0.19 |
| Chronic obstructive pulmonary disease | 5 (11.6) | 62 (10.7) | 1.00 |
| Systolic blood pressure (mm Hg) | 125 (16.5) | 135 (19.4) | < 0.001 |
| Systolic blood pressure > 140 mm Hg | 3 (7.0) | 189 (32.6) | < 0.001 |
| Diastolic blood pressure (mm Hg) | 74.4 (12.5) | 76.5 (12.9) | 0.37 |
| LDL-c (mg/dl) | 80.5 (34.8) | 83.9 (36.9) | 0.61 |
| LDL-c > 100 mg/dl | 8 (18.6) | 82 (14.2) | 1.00 |
| Hemoglobin A1c (%) | 6.7 (1.3) | 6.9 (1.6) | 0.35 |
| Statin MPR | 65.9 (28.1) | 65.1 (28.6) | 0.86 |
| Statin MPR < 50% | 7 (16.3) | 136 (23.5) | 0.40 |
| Antihypertensive medication MPR | 66.4 (25.3) | 62.5 (25.5) | 0.34 |
| Antihypertensive MPR < 50% | 9 (20.9) | 140 (24.2) | 0.81 |

Continuous variables presented as mean (SD); categorical variables presented as n (%). ASCVD, atherosclerotic cardiovascular disease; MPR, medication possession ratio (days with medication supply divided by total days)

**Supplemental Figure 1:** Proportion of participants submitting blood pressure measurements by week


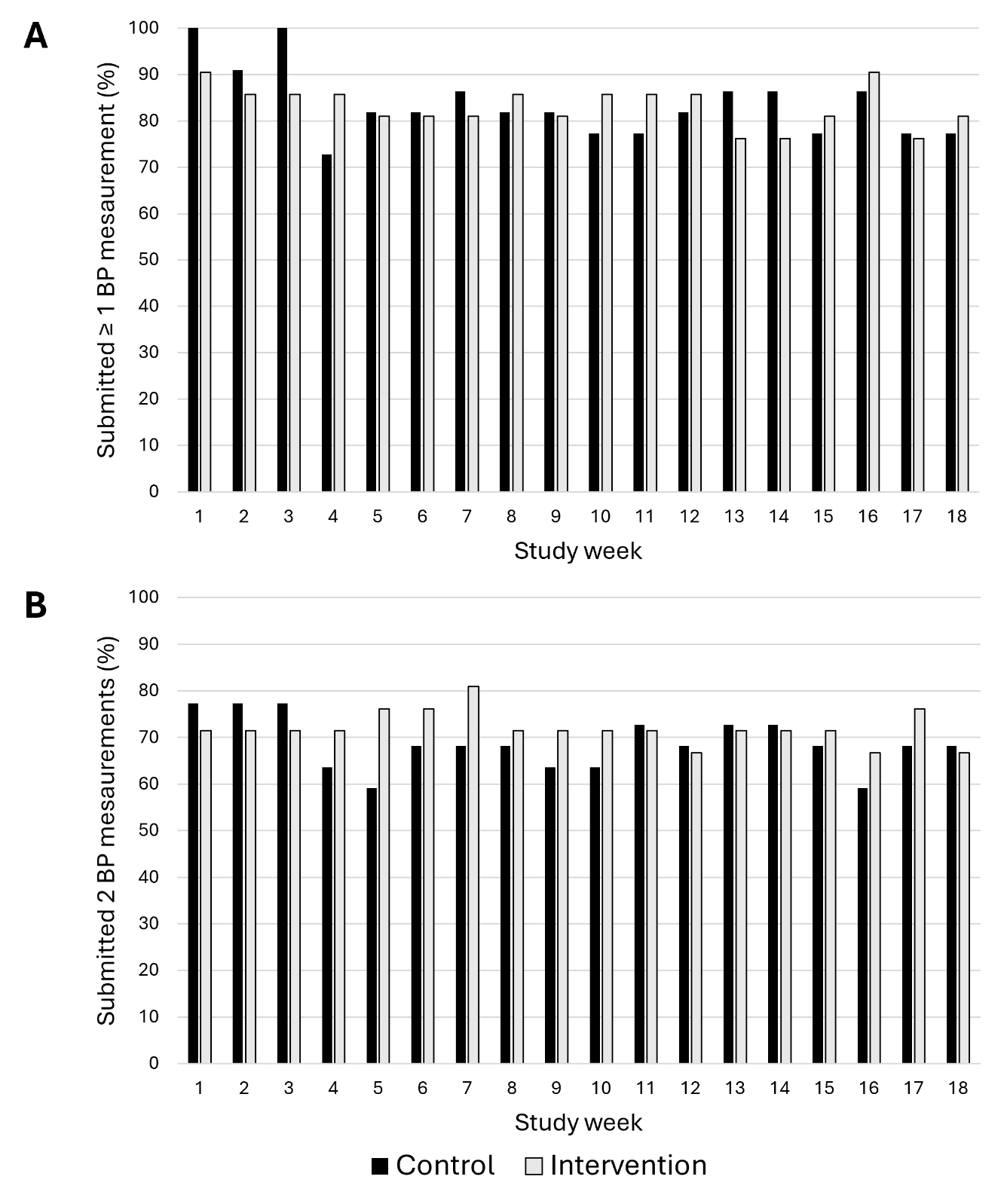


Figure depicts the proportion of patients by arm who submitted at least one (panel A) and two (panel B) blood pressure measurements in each study week. Intervention arm participants submitted at least one blood pressure measurement on 83.1% of participant-weeks; control participants submitted at least one blood pressure measurement on 83.6% of participant-weeks. BP, blood pressure.
